# Diagnostic performance of artificial intelligence models for pulmonary nodule classification: a multi-model evaluation

**DOI:** 10.1007/s00330-025-11845-1

**Published:** 2025-07-25

**Authors:** Sarah K. Herber, Lukas Müller, Daniel Pinto dos Santos, Tobias Jorg, Fabio Souschek, Tobias Bäuerle, Sebastian Foersch, Christian Galata, Peter Mildenberger, Moritz C. Halfmann

**Affiliations:** 1 Department of Diagnostic and Interventional Radiology, University Medical Center of the Johannes Gutenberg-University Mainz, Mainz, Germany; 2 Institute of Pathology, University Medical Center of the Johannes Gutenberg-University Mainz, Mainz, Germany; 3 Department of Thoracic Surgery, University Medical Center of the Johannes Gutenberg-University Mainz, Mainz, Germany

**Keywords:** Artificial intelligence, Lung cancer, Pulmonary nodules, Computed tomography, Diagnostic accuracy

## Abstract

**Objectives:**

Lung cancer is the leading cause of cancer-related mortality. While early detection improves survival, distinguishing malignant from benign pulmonary nodules remains challenging. Artificial intelligence (AI) has been proposed to enhance diagnostic accuracy, but its clinical reliability is still under investigation. Here, we aimed to evaluate the diagnostic performance of AI models in classifying pulmonary nodules.

**Materials and methods:**

This single-center retrospective study analyzed pulmonary nodules (4–30 mm) detected on CT scans, using three AI software models. Sensitivity, specificity, false-positive and false-negative rates were calculated. The diagnostic accuracy was assessed using the area under the receiver operating characteristic (ROC) curve (AUC), with histopathology serving as the gold standard. Subgroup analyses were based on nodule size and histopathological classification. The impact of imaging parameters was evaluated using regression analysis.

**Results:**

A total of 158 nodules (*n* = 30 benign, *n* = 128 malignant) were analyzed. One AI model classified most nodules as intermediate risk, preventing further accuracy assessment. The other models demonstrated moderate sensitivity (53.1–70.3%) but low specificity (46.7–66.7%), leading to a high false-positive rate (45.5–52.4%). AUC values were between 0.5 and 0.6 (95% CI). Subgroup analyses revealed decreased sensitivity (47.8–61.5%) but increased specificity (100%), highlighting inconsistencies. In total, up to 49.0% of the pulmonary nodules were classified as intermediate risk. CT scan type influenced performance (*p* = 0.03), with better classification accuracy on breath-held CT scans.

**Conclusion:**

AI-based software models are not ready for standalone clinical use in pulmonary nodule classification due to low specificity, a high false-negative rate and a high proportion of intermediate-risk classifications.

**Key Points:**

***Question*** *How accurate are commercially available AI models for the classification of pulmonary nodules compared to the gold standard of histopathology?*

***Findings*** *The evaluated AI models demonstrated moderate sensitivity, low specificity and high false-negative rates. Up to 49% of pulmonary nodules were classified as intermediate risk.*

***Clinical relevance*** *The high false-negative rates could influence radiologists’ decision-making, leading to an increased number of interventions or unnecessary surgical procedures.*

**Graphical Abstract:**

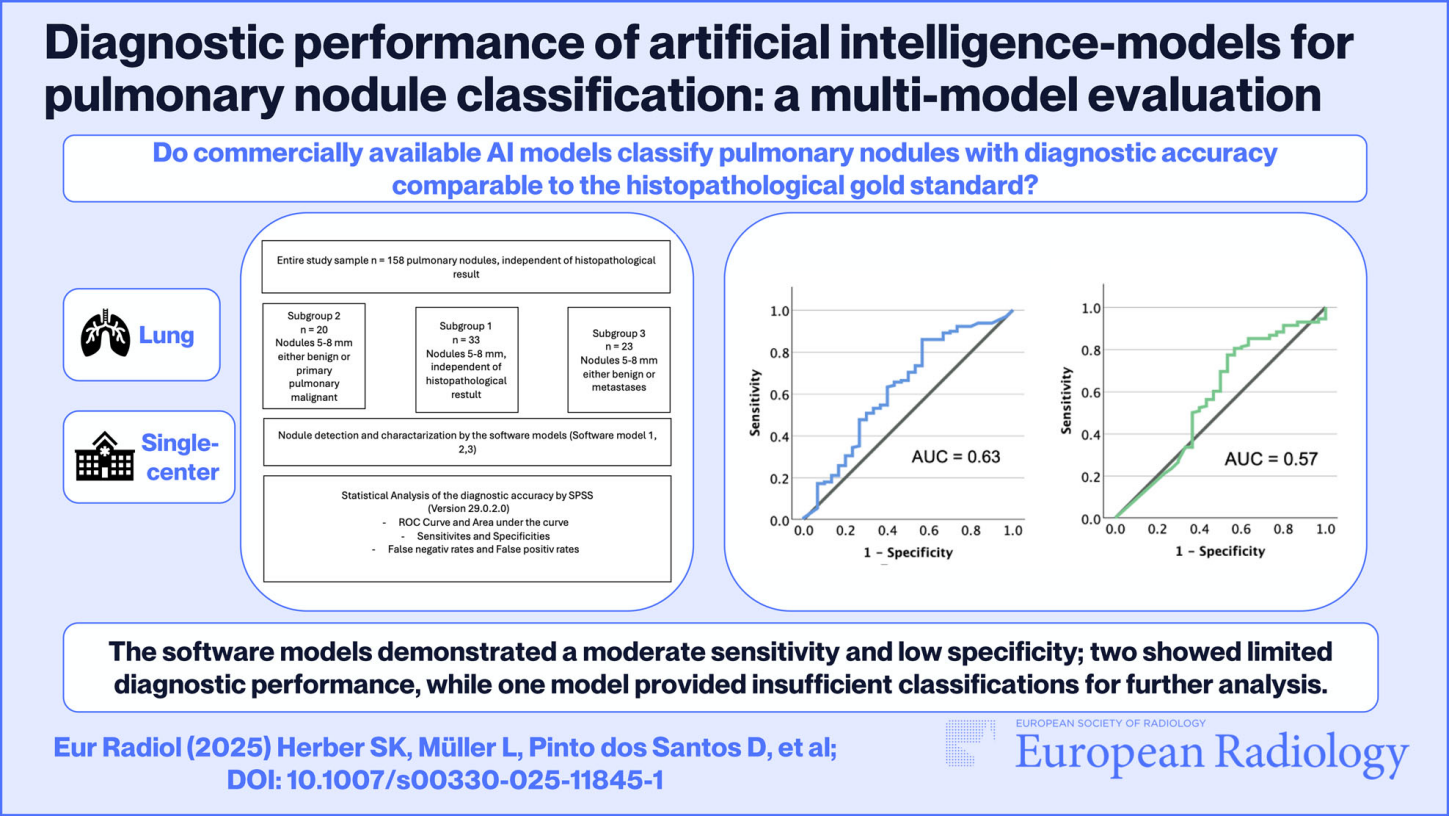

## Introduction

Lung cancer mortality outranks all other cancer types by a two-fold margin [1]. The poor 5-year survival rate is often associated with the late detection of lung cancer at advanced, incurable stages [2]. Prognosis is dependent on the stage of diagnosis [3]. Therefore, improving patient outcomes requires the early diagnosis of malignant pulmonary nodules [4].

This has fueled the development of high-resolution CT scanners, potentially able to detect even small pulmonary nodules [5]. However, parallel to the higher resolution of these scanners, the detection rate of pulmonary nodules in thoracic CT scans has also significantly risen, taking a toll on specificity [6]. Additionally, with advancing implementations of lung cancer screening, the number of pulmonary nodules requiring classification in thoracic CT scans is expected to increase significantly.

In the past, various models have been developed with the goal to predict malignancy risk, often associated with the size, morphologic characteristics and the growth rate of nodules [7]. In this context, AI models have recently become recognized for their potential to optimize such follow-up protocols by automating nodule detection, reducing false positives and enhancing risk prediction, thereby improving patient outcomes [6]. In addition to the detection and localization of pulmonary nodules, the AI software models are also designed to classify them as benign or malignant, further supporting clinical decision-making [8].

However, despite these advancements, the clinical adoption of AI in pulmonary nodule assessment remains challenging [9]. This has been attributed to limited generalizability of studies, the lack of transparency in their decision-making process and insufficient data on their impact on radiologists’ decisions and patient outcome [10]. Therefore, this study aimed to evaluate the diagnostic accuracy of three AI models for the classification of pulmonary nodules against the gold standard of histopathology.

## Materials and methods

The local ethics committee approved the protocol of this retrospective study with a waiver for informed consent (Ethics committee of Rhineland Palatinate, Germany, reference number 2023-16965).

### Study sample

In this single-center study, patients who underwent CT-guided lung biopsy or surgical resection at a tertiary care hospital between 2018 and 2024 were retrospectively identified from the institutional picture archiving and communications system (PACS). The inclusion criteria were as follows: age above 18 years, CT scan covering the entire thoracic cavity with a maximal slice thickness of 4 mm, presence of at least one pulmonary nodule measuring between 4 and 30 mm in long-axis diameter and histopathological results of an individually identifiable pulmonary nodule. Exclusion criteria included follow-up CT scans of previously known pulmonary nodules, pulmonary nodules smaller than 4 mm or larger than 30 mm.

A board-certified radiologist with more than 20 years of experience (P.M.) confirmed the presence of pulmonary nodules between 4 and 30 mm in size. For further analysis, pulmonary nodules were additionally split into subgroups to address challenging clinical scenarios where follow-up CT scans, CT-guided biopsies or resection are frequent [11]. Pulmonary nodules were categorized into 3 subgroups based on their size and histopathological outcome: (1) all nodules between 5 and 8 mm, independent of histopathological results, (2) nodules between 5 and 8 mm and either benign or primary pulmonary malignancies as histopathological result and (3) nodules between 5 and 8 mm and either benign or metastases of non-pulmonary primaries as histopathological result. The differentiation between metastases and non-pulmonary primaries was made because the AI models were primarily trained to detect and categorize primary lung cancer, which exhibits different morphological characteristics compared to metastases [12].

### AI models

All CT scans were separately sent directly from PACS to the three AI models. The following commercial software solutions (listed in alphabetical order) were used: ADVANCE Chest CT (Version 2.2.1), Contextflow GmbH; InferRead® CT Lung, Infervision Medical Technology (Version 1.0.1.1); and Rayscape Lung CT (Version 2-1-174-1.278-2.153), RAYSCAPE Mindfully Technologies. All models were hosted on premises at the hospital and provided results fully automatically without the need for user interaction. In this study, the software models are described in non-alphabetical order as software model 1, software model 2, and software model 3.

All models are designed to detect pulmonary nodules, measure their size and volume and calculate a probability of malignancy. They automatically identified and evaluated various types of pulmonary nodules (solid, mixed, and ground glass) in CT scans. Malignancy risk was classified as high, intermediate or low by each model applying different proprietary scoring systems. As the categorization of malignancy risk was determined individually by each software model, the thresholds varied between the models. Each software vendor applied individual thresholds to derive the three risk categories, which were not adjusted to local prevalences. Model 1 provided a continuous variable from 0 to 100% with higher values indicating a higher probability of malignancy and a threshold value of < 19% as benign, 19–62% intermediate risk, and > 62% as malignant. Model 2 provided a continuous variable from 0.0 to 1.0 with higher values indicating higher probability of malignancy and a threshold value of < 0.1 as benign, 0.1–0.9 as intermediate and > 0.9 as malignant. For model 3, no numerical thresholds were available; instead, the software produced categorical outputs (“low,” “medium,” “high”) without specifying a continuous variable.

Software models 2 and 3 were limited to detecting and evaluating pulmonary nodules measuring between 3 and 30 mm. Additionally, software model 2 calculated a malignancy risk score only for the seven largest detected pulmonary nodules.

### Statistical analysis

All statistical analyses were performed using dedicated statistical software (SPSS, Version 29.0.2.0, IBM Corporation). Continuous variables were tested for the assumption of a normal distribution using the Shapiro–Wilk test. Based on the respective results, they are reported as mean with standard deviation or median with interquartile range. Categorical variables are reported as absolute frequencies with respective proportions. Chi-squared tests were utilized to compare categorical variables across groups. For comparison of the subgroups, which had smaller sample sizes, Fisher’s exact tests were applied. For non-normally distributed data, the Mann–Whitney U test was used. A *p*-value of < 0.05 was considered statistically significant.

The diagnostic accuracy was assessed by receiver operating characteristic (ROC) curves with respective areas under the curve (AUC) based on the raw data of the malignancy scores of each model. Sensitivities and Specificities were derived from ROC curves based on the highest Youden’s indices. The histopathological results served as ground truth and were transformed into a numerical value, which served as a state variable, while the malignancy scores functioned as the test variable. Additionally, the false-negative rate (FNR) and false-positive rate (FPR) were calculated from cross tables based on the dichotomous interpretation of the malignancy scores as recommended by the models’ vendors.

The impact of potential confounders and their influence on the results was assessed by univariable regression analysis. The dependent variables were the general classification (malignant/benign vs. intermediate) and the correct classification (correct vs. false). The independent variables included patient characteristics and CT scan-related characteristics such as sex, age, CT scanner type, use of contrast agent, slice thickness and type of histopathology sampling. To quantify the impact on the diagnostic accuracy, odds ratios (OR) with a 95% confidence interval (CI) were calculated for each variable.

## Results

### Patient characteristics

The entire study sample included 152 patients (41.5% female, mean age 64 ± 15 years) with a total of 158 pulmonary nodules. A total of 30/158 (18.9%) were histopathologically classified as benign, 67/158 (42.4%) as primary pulmonary malignancies, and 61/158 (38. 6%) as metastases of cancers of different origin.

There were three distinct subgroups, each with a nodule size between 5 and 8 mm, which were stratified based on underlying histopathology: (1) Subgroup 1 included 30 patients with a total of 33 pulmonary nodules, independent of histopathology. (2) Subgroup 2 consisted of 19 patients with 20 benign or primary pulmonary malignant nodules. (3) Subgroup 3 included 20 patients with 23 pulmonary nodules, which were either benign or metastases of non-pulmonary primaries pulmonary nodules. Further patient and scan characteristics can be found in Table 1.

**Table 1:** Characteristics of the study sample

	Study sample	Subgroups		
		1	2	3
Patients				
Number of patients	152	30	19	20
Female sex	63 (41.5%)	17 (56.7%)	11 (57.9%)	10 (50.0%)
Mean age (years)	64 ± 15	58 ± 18	61 ± 18	56 ± 19
Histopathology				
Benign	30 (19.0%)	10 (30.3%)	10 (50.5%)	10 (43.4%)
Primary malignancy	67 (42.4%)	10 (30.3%)	10 (50.0%)	n/a*
Metastasis	61 (38.6%)	13 (39.4%)	n/a*	13 (56.5%)
Type of sampling				
Surgical	111 (70.3%)	31 (93.9%)	19 (95.5%)	22 (95.7%)
CT biopsy	47 (29.7%)	2 (6.1%)	1 (5.0%)	1 (4.3%)
CT scan				
Mean nodule size (mm)	14.7 ± 7.0	6.8 ± 1.1	6.3 ± 1.0	7.0 ± 1.0
Median slice thickness (mm)	1.0 (1.0–3.0)	1.0 (1.0–3.0)	1.0 (1.0–3.0)	1.0 (1.0–3.0)
Contrast media usage	94 (59.5%)	13 (39.4%)	7 (35.0%)	8 (34.8%)
Breath-held during scan	135 (85.4%)	22 (66.7%)	15 (75.0%)	13 (56.5%)
Location of CT scan				
Local	120 (75.9%)	27 (81.8%)	16 (80.0%)	19 (82.6%)
External	38 (24.1%)	6 (18.2%)	4 (20.0%)	4 (17.4%)
Scanner manufacturer				
Philips	97 (61.4%)	25 (75.8%)	14 (70.0%)	18 (78.3%)
Siemens	46 (29.1%)	6 (18.2%)	4 (20.0%)	4 (17.4%)
General Electric	7 (4.4%)	2 (6.1%)	2 (10.0%)	1 (4.3%)
Toshiba	6 (3.8%)	0 (0.0%)	0 (0.0%)	0 (0.0%)
Canon	2 (1.3%)	0 (0.0%)	0 (0.0%)	0 (0.0%)

All data are absolute frequencies with respective proportions unless otherwise specified

* Not applicable due to selection criteria for the respective subgroups

### Nodule detection and classification

Out of 158 pulmonary nodules, software model 1 detected 150 (94.9%) and categorized 141 (89.2%), with 106 (67.1%) classified as benign or malignant. Model 2 detected 124 (78.5%) and categorized 114 (72.2%), with 60 (38.0%) classified as benign or malignant based on the interpretation of the malignancy scores as recommended by the models’ vendors. Model 3 detected 122 (84.2%) and categorized 67 (42.4%), but only 6 (3.8%) were classified as benign or malignant based on the interpretation of the malignancy scores as recommended by the model’s vendor. Detailed results and subgroup analyses can be found in Table 2.

**Table 2:** Detection and classification

	Model 1	Model 2	Model 3	*p*-value
Entire study sample (*n* = 158)				
Detected nodules	150 (94.9%)	124 (78.48%)	133 (84.2%)	0.28
Classified nodules	141 (88.0%)	114 (72.2%)	67 (42.4%)	< 0.001
Benign	30 (19.0%)	20 (12.7%)	1 (0.6%)	< 0.001
Intermediate	35 (22.2%)	48 (30.4%)	61 (38.6%)	0.03
Malignant	76 (48.1%)	46 (29.1%)	5 (3.2%)	< 0.001
Subgroup 1 (*n* = 33)				
Detected nodules	32 (97.0%)	30 (90.9%)	30 (90.9%)	0.96
Classified nodules	32 (97.0%)	30 (90.9%)	16 (48.5%)	0.05
Benign	20 (60.6%)	2 (6.1%)	0 (0.0%)	< 0.001
Intermediate	8 (24.2%)	15 (45.5%)	16 (48.5%)	0.23
Malignant	4 (12.1%)	13 (39.4%)	0 (0.0%)	< 0.001
Subgroup 2 (*n* = 20)				
Detected nodules	20 (100.0%)	18 (90.0%)	17 (85.0%)	0.88
Classified nodules	20 (100.0%)	18 (90.0%)	10 (50.0%)	0.17
Benign	14 (70%)	9 (45.0%)	0 (0.0%)	< 0.001
Intermediate	4 (20.0%)	9 (45.0%)	10 (0.0%)	0.26
Malignant	2 (10.0%)	0 (0.0%)	0 (0.0%)	0.14
Subgroup 3 (*n* = 23)				
Detected nodules	22 (95.6%)	21 (90.3%)	20 (86.9%)	0.95
Classified nodules	22 (95.6%)	21 (90.3%)	9 (39.1%)	0.05
Benign	2 (8.7%)	2 (8.7%)	0 (0.0%)	0.37
Intermediate	5 (21.7%)	10 (43.5%)	9 (39.1%)	0.42
Malignant	15 (65.2%)	9 (39.1%)	0 (0.0%)	< 0.001

All data are absolute frequencies with respective proportions

For some pulmonary nodules, which were detected by the software models, no malignancy risk prediction was available. In this instance, the software vendors did not provide an explicit reason for the absence of a calculated malignancy risk. A possible but unconfirmed explanation from the software vendors is that case-specific image characteristics may have prevented risk estimation; however, no detailed explanation was disclosed. As a result, the cause for missing categorization remains unknown.

### Diagnostic accuracies

Due to the small number of classified pulmonary nodules as either benign or malignant, software model 3 did not provide a statistically reliable basis for further evaluation. Therefore, the model was excluded from further analysis. The potential reasons for the failure of software model 3 to provide a malignancy risk classification remain hypothetical, as no definitive explanation was provided by the software vendor. To our best knowledge, technical limitations such as limited image quality, cases outside the model’s training range (e.g., atypical shape or localization of pulmonary nodules), slice thickness or differences between breathing and non-breathing CT scans may have influenced the model’s performance.

**Fig. 1:**
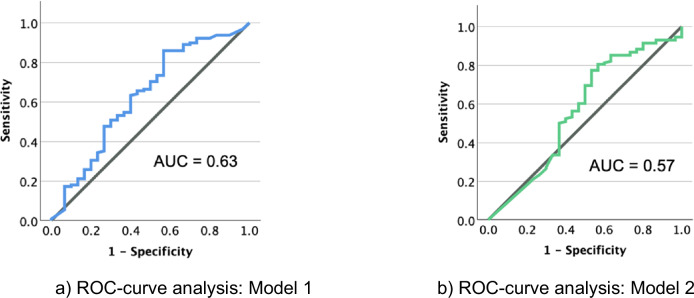
ROC-curve analysis of the entire study sample (**a** = model 1, **b** = model 2)

For the entire study sample, the remaining software models demonstrated a moderate sensitivity (53.1% vs. 70.3%, *p* = 0.005) and a moderate to low specificity (66.7% and 46.7%, *p* = 0.12), respectively. The FNR was moderate, with values of 22.4% vs. 25.5% for both models (*p* = 0.65). Both models showed comparable FPR (52.4% vs. 45.5%, *p* = 0.49). The AUC values reached 0.63 vs. 0.57, respectively. The corresponding ROC curves are shown in Fig. 1.

**Fig. 2:**
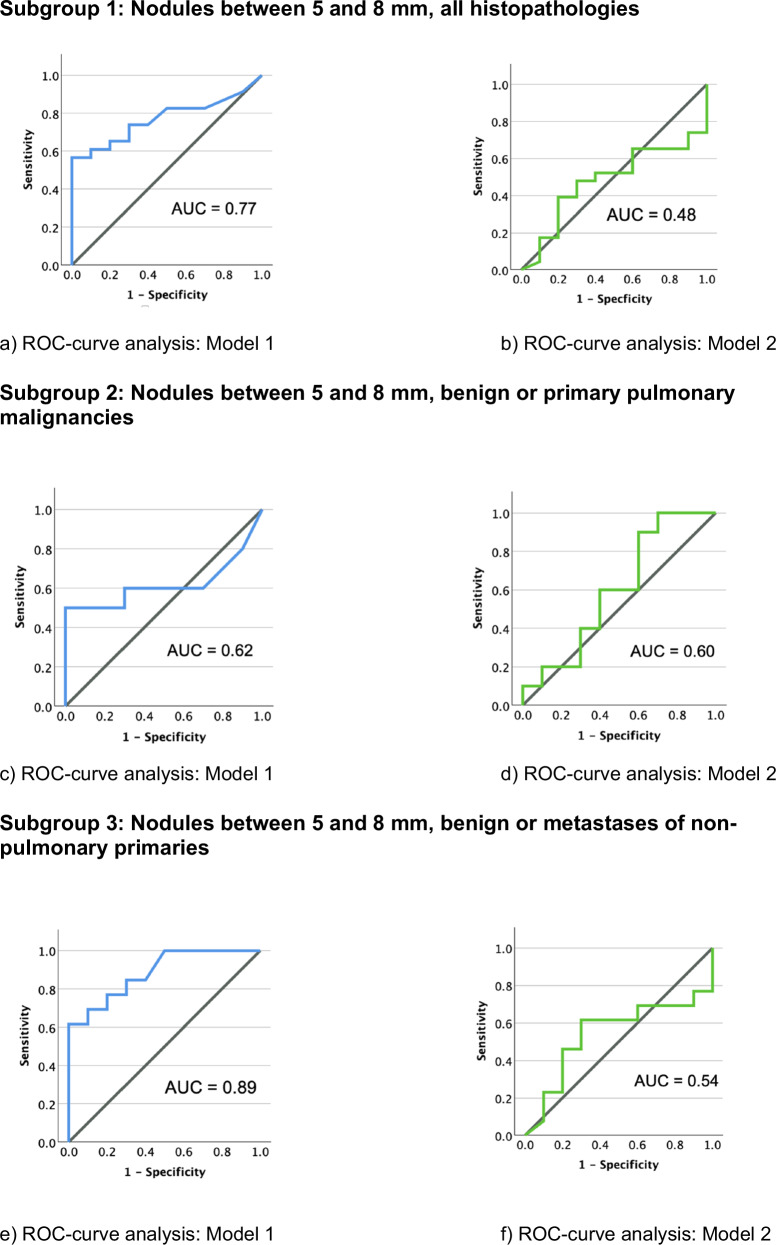
ROC-curve analysis of the subgroups (**a**, **c**, **e** = model 1; **b**, **d**, **f** = model 2)

In subgroup 1, both software models showed a lower sensitivity than in the entire study sample (47.8% and 52.2%, respectively). In comparison to the entire study sample, the models demonstrated remarkably higher specificity, reaching 100.0% vs. 60.0% but simultaneously higher FNR of 73.3% and 83.3%, respectively. ROC analysis showed statistically significant differences in the AUC values for subgroup 1 (0.77 vs. 0.48). Figure 2 illustrates the corresponding ROC curves and AUC values.

In subgroup 2, statistical analysis showed no significant differences in sensitivity between the two software models (50.0% vs. 60.0%, *p* = 1.00). Similar to subgroup 1, model 1 demonstrated a high specificity of 100.0% (vs. 60.0%) and a high FNR of 71.4%. and 100.0%, respectively. ROC analysis showed comparable AUC values for subgroup 2 (0.62 vs. 0.60). The ROC curves and AUC values are presented in Fig. 2.

For subgroup 3, both models showed moderate sensitivity with a minimum of 61.5% (*p* = 0.69). Similar to the other subgroups, the two models demonstrated a high specificity of 100.0% and 70.0%, respectively. The statistical analysis revealed a high FNR of 71.4–75.0% and a low FPR (0.0%) for both software models. The AUC value showed a minimum of 0.54. Figure 2 presents the corresponding ROC curves and AUC values.

Detailed results of the diagnostic accuracies of both models in the entire study sample and the respective subgroups are summarized in Table 3. Interestingly, due to the exclusion of the intermediate risk nodules, sensitivities and specificities were markedly lower when using the dichotomous cut-offs provided by the vendors (Supplementary Table 1).

**Table 3:** Diagnostic accuracy

	Model 1	Model 2	*p*-value
Entire study sample			
Youden Index	0.198	0.17	
Cutoff value	71.47	0.61	
Sensitivity	53.1%	70.3%	0.005
Specificity	66.7%	46.7%	0.12
FNR	22.4%	25.5%	0.65
FPR	52.4%	45.5%	0.49
Subgroup 1			
Youden Index	0.48	0.12	
Cutoff value	28.11	0.35	
Sensitivity	47.8%	52.2%	0.13
Specificity	100.0%	60.0%	1.00
FNR	73.3%	83.3%	0.42
FPR	0.0%	0.0%	1.00
Subgroup 2			
Youden Index	0.40	0.20	
Cutoff value	6.27	0.22	
Sensitivity	50.0%	60.0%	1.0
Specificity	100.0%	60.0%	0.016
FNR	71.4%	100.0%	0.03
FPR	0.0%	0.0%	1.00
Subgroup 3			
Youden Index	0.62	0.32	
Cutoff value	17.07	0.44	
Sensitivity	61.5%	61.5%	0.69
Specificity	100.0%	70.0%	0.026
FNR	75.0%	71.4%	0.77
FPR	0.0%	0.0%	1.00

*FNR* false-positive rate, *FPR* false-negative rate

In addition to the main analysis focusing on pulmonary nodules between 4 and 30 mm and the subgroup analysis focusing on pulmonary nodules measuring 5–8 mm, diagnostic performance metrics were also calculated for the nodules between 9 and 30 mm. These results are shown in Supplementary Table 2 and Supplementary Fig. 1.

### Factors influencing classification

The comparison of the independent variables of sex, location of the CT scan, breath-hold vs. free-breathing technique, use of contrast media, slice thickness and type of sampling (surgical resection vs. CT-guided biopsy) showed statistically significant differences in regard to the correct classification in benign or malignant nodules for the slice thickness (*p* = 0.02) for one model and the type of sampling for the other (*p* = 0.01).

The univariable regression analysis showed that the use of breath-hold CT scans was associated with an increased probability of correct classification of pulmonary nodules as benign or malignant for one model (*p* = 0.03). For other potential confounders, no statistically significant effect on the correct classification of nodules was found. The detailed results of the possible influencing factors on the results of software models 1 and 2 are summarized in Table 4.

**Table 4:** Regression analysis for the correct classification of pulmonary nodules

Variable	Model 1		Model 2	
	Univariate hazard ratio	*p*-value	Univariable hazard ratio	*p*-value
Sex	1.08	0.86	0.62	0.21
Age	1.74	0.23	0.91	0.88
Slice thickness	0.79	0.6	0.45	0.23
Type of sample	1.73	0.25	0.88	0.86
CT location	0.49	0.16	0.57	0.34
Use of contrast media	0.9	0.82	0.53	0.3
Scanner manufacturer	1.97	0.24	0.43	0.03

Categorical variables were encoded as dummy variables

In the analysis of potential of factors influencing the ability of a model to dichotomously classify a pulmonary nodule, slice thickness and the type of sampling (biopsy vs. surgical extraction) were identified as statistically significant. Further details can be found in Supplementary Table 3.

## Discussion

This study investigated three commercially available AI models for the detection and characterization of pulmonary nodules compared to histopathological ground truth. The main findings can be summarized as follows: models showed moderate sensitivities but low specificities. The diagnostic accuracies for two of the software models were limited, while one software failed to classify the majority of nodules as either benign or malignant, thus barring further analysis.

The increasing detection of pulmonary nodules in routine CT scans presents diagnostic challenges, particularly for nodules < 8 mm, which lack clear image features and have high biopsy failure rates. This often leads to repeated follow-up scans, increasing radiation exposure, financial burden and patient anxiety [13]. Furthermore, false-positive findings result in unnecessary invasive procedures and healthcare strain, while false negatives delay diagnosis, giving patients a false sense of security and potentially postponing treatment [14]. Accurate risk-stratified assessment is therefore crucial to improve early cancer detection while minimizing unnecessary interventions [15].

For lung cancer diagnosis, AI has been proven to enhance diagnostic accuracy and efficiency in early detection using CT scans of the thorax and X-rays and reducing false positives [16]. Additionally, it has been shown that the use of AI in lung cancer detection can significantly increase the sensitivity of radiologists and reduce the false-positive rate for pulmonary nodules > 5 mm [17]. Moreover, when AI models are applied as a second reader alongside radiologists, sensitivity can be further improved by identifying nodules missed by radiologists. Studies have shown that AI systems can detect pulmonary nodules not identified by radiologists, while radiologists may also detect nodules overlooked by AI, demonstrating the complementary potential of combining human expertise with AI systems [18]. In lung cancer screening, AI could be integrated to support the detection of pulmonary nodules and risk stratification [19]. Further, AI can characterize different types of pulmonary nodules (spiculated, solid, partially solid) with a comparable performance to radiologists [20]. For malignancy prediction, AI software models helped to achieve better diagnostic accuracy [21].

Multiple studies have demonstrated high accuracy in classifying lung cancer types using convolutional neural networks (CNNs) in lung cancer classification [22]. Quanyang et al compared two automated classification models for identifying the histopathological types of lung cancer on CT scans, with accuracies of 72.0–79.0% and AUC values of 0.79–0.82 [23]. Yoo et al evaluated an AI model specifically designed to detect malignant nodules in chest radiographs. The study reported higher sensitivity and specificity compared to radiologists, demonstrating the potential to improve lung cancer detection in X-ray imaging [24].

Further, the performance of AI in classifying pulmonary nodules in benign or malignant was compared to radiologists by Hu et al, who showed a higher sensitivity and specificity for the software models than the radiologists [25]. Hendrix et al investigated the detection and classification of pulmonary nodules. Their AI model achieved high sensitivity up to 96.6% for benign nodules, primary lung cancer and lung metastases. However, the results of their AI model were validated by radiologist assessments and the national cancer registry [26]. In contrast, our study directly compared the classification performance of three AI models against the histopathological gold standard, ensuring an objective reference for benign and malignant classification. This approach provided a more definitive evaluation of the diagnostic accuracy of AI models, as it eliminates the potential bias from radiologists.

The three software modules of our study demonstrated moderate sensitivity, a low specificity, a moderate false-negative rate, and low false-positive rates. This indicates that all three software modules of our study were relatively successful in identifying malignant nodules but had difficulties with the correct classification of benign pulmonary nodules, resulting in a high rate of false positives. A high false-positive rate could lead to overtreatment, including unnecessary invasive procedures.

The diagnostic performance of the software models changed when analyzing subgroups based on the size of pulmonary nodules and histopathological classification. Sensitivity decreased in nearly all subgroups, which indicates that the software models struggled with the accurate classification of pulmonary nodules when divided into smaller, more homogeneous subgroups. This finding suggests that smaller pulmonary nodules may be more challenging to classify for the software models.

In contrast, the specificity increased across all three subgroups, indicating improved performance in correctly identifying benign nodules. The wide range of values highlights inconsistencies in the software’s reliability.

The increase in FNR is clinically concerning, as this could result in malignant pulmonary nodules being overlooked and therefore delayed diagnosis and treatment with subsequent negative downstream effects on patients’ outcome [27].

Significant differences were identified regarding the influence of independent variables such as slice thickness and the type of sampling (biopsy vs. surgical resection). A likely explanation lies in the effect of these parameters on image quality. Increased slice thickness leads to greater volume averaging, resulting in blurred nodule margins that may be misinterpreted by the software models. Similarly, differences in the scan acquisition may induce variations in lesion characteristics that affect automated evaluation. Additionally, our analyses showed a significant impact of the type of CT scan (breath hold vs. free breathing) on the diagnostic performance of one software model. A possible explanation for this is that the software models were primarily trained on breath-held CT scans, thus resulting in a reduced performance on CT scans acquired in free breathing. Another contributing factor could be suboptimal breath holding, which may introduce motion artifacts such as slight blurring of anatomical structures, potentially leading to artificially increased density measurements and subsequent misclassifications.

The findings underscore the importance of carefully assessing AI tools when using them outside their original training or conception, which was recently corroborated in a study by Li et al [28]. The study demonstrated that the performance of AI models varies depending on the clinical application and, similar to our results, models had relatively low diagnostic accuracy (AUC up to a maximum of 0.6) in the assessment of biopsied pulmonary nodules. The authors therefore concluded that AI models for pulmonary nodule risk assessment are not yet suitable for use as a standalone diagnosis in clinical routine.

However, the absence of an influence of the other analyzed potential confounders in our study suggests a certain level of robustness. While the confounder analyses highlighted factors that may have influenced the performance of the software models, non-classified pulmonary nodules (categorized with an intermediate malignancy risk) represented the most severe limitation of the software models from a clinical viewpoint. Depending on the model, up to half of all pulmonary nodules were not classified as malignant or benign based on the vendors’ dichotomous interpretation guidelines. Of these intermediate risk nodules, the majority (44.4–88.5%) showed malignant histopathology. These results could potentially lead to a misdiagnosis of pulmonary nodules and thus negatively impact downstream management. Therefore, it is important to benchmark the often heavily training dataset-dependent and prevalence-adjusted models to real-world data in order to find optimal cutoff values for individualized risk prediction.

This study has limitations. It included a consecutive patient collective, which involves different scanner manufacturers, varying slice thicknesses and different acquisition techniques. Despite its reflection of real-world conditions, this may have introduced selection bias, thereby reducing the models’ ability to extract distinguishing features between benign and malignant nodules, which likely influenced the AUC values. Additionally, the unequal distribution of benign and malignant nodules in the dataset may have influenced the diagnostic accuracies.

Another limitation of this study is the retrospective design, with patient inclusion being based on available histopathology and resulting inability to report Free-Response Receiver Operating Characteristic (FROC). In cases with multiple pulmonary metastases, only one pulmonary nodule was biopsied or surgically resected for histopathological confirmation, while additional nodules were not systematically evaluated. As a result, the true dignity of these (non-sampled) pulmonary nodules remains unknown. Assuming all non-sampled nodules to be benign (as would be required to calculate FROC curves) could introduce substantial bias. Therefore, the sensitivity and specificity were calculated based on nodules with confirmed histology, which may, in turn, limit the comprehensive performance assessment of the models. Future studies with systematic histological assessment or follow-up of all detected nodules are needed to address this limitation.

One further limitation is that the software models were trained on incidental pulmonary nodules rather than being explicitly developed for high-risk populations such as the study sample. As a result, their performance may not optimally transfer to this specific patient group, potentially contributing to the observed results.

It is important to note that the sample size in this study was relatively small for the image classification task, which may have limited the models’ performance and generalizability. In addition, the sample size in the subgroups was limited, which may have further restricted the generalizability of the findings, as imbalanced distributions could have introduced bias, complicating the interpretation of the results. These limitations emphasize the need for larger datasets and more balanced subgroup distributions in future multicenter studies to enable more robust model training and support more reliable conclusions.

Additionally, in clinical practice, the characterization of pulmonary nodules is based on the evaluation of multiple variables such as patient history and other risk factors, especially for nodules with an intermediate malignancy risk. Clinical predictors are necessary to assess the risk of these nodules using the analyzed AI models. However, in this study, the software models assessed the pulmonary nodules without contextual information, relying solely on imaging-based classification. This may be considered another limitation, as it does not fully reflect real-world clinical decision-making. Future studies may assess the impact of multimodal models incorporating clinical predictors as additional variables during analysis.

## Conclusion

Currently, artificial intelligence applications for the prediction of the malignancy risk of pulmonary nodules show potential but are not yet ready to serve as standalone tools in clinical routine due to limited diagnostic accuracy. While limitations apply regarding the retrospective study design and sample size, the high false-negative rate of the AI models poses the risk of influencing radiologists’ decision-making. Further research with larger, more diverse and study samples is needed to confirm these findings.

## Supplementary information

Supplementary information

## Abbreviations

AI: Artificial intelligence
AUC: Area under the curve
CI: Confidence interval
CT: Computed tomography
FNR: False negative rate
FPR: False positive rate
FROC: Free-response receiver operating characteristics
OR: Odds ratio
PACS: Picture Archiving and Communications System
ROC: Receiver operating curve
X-rays: X-radiation
