# Supplementary material for: Diagnostic performance of artificial intelligence models for pulmonary nodule classification: a multi-model evaluation
Source: Eur Radiol. 2025 Jul 25;36(1):537–47. doi: 10.1007/s00330-025-11845-1 (PMC12712079; doi:10.1007/s00330-025-11845-1)
Supplement: Supplementary file 1 — Supplementary information [file 330_2025_11845_MOESM1_ESM.pdf]

Diagnostic Performance of Artificial Intelligence-Models for Pulmonary Nodule Classification: A Multi-Model Evaluation

ELECTRONIC SUPPLEMENTARY MATERIAL

Supplemental Figure1, Subgroup 4: Nodules between 9 – 30 mm, all histopathologies

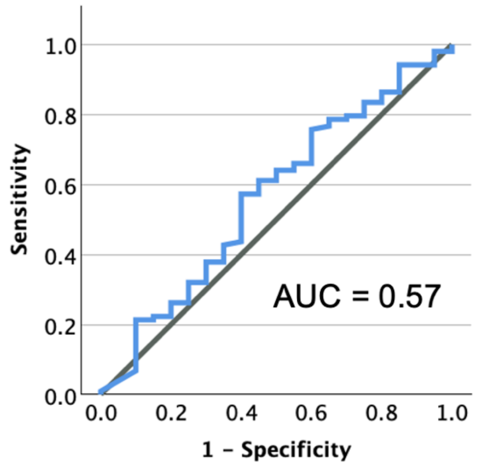

a) ROC-curve analysis: Model 1

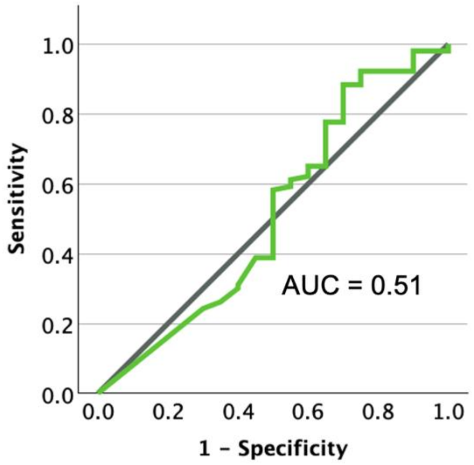

b) ROC-curve analysis: Model 2

Supplemental Figure 1: ROC-curve analysis, subgroup 4

Supplemental Table 1: Diagnostic Accuracy, dichotomous cut-offs provided by the vendors

	Model 1	Model 2	*p* – value
Entire study sample			
Sensitivity	77.7 %	74.6 %	0.80
Specificity	47.6 %	55.5 %	0.44
Subgroup 1			
Sensitivity	26.7 %	16.7 %	0.13
Specificity	100.0 %	100.0 %	1.00
Subgroup 2			
Sensitivity	28.6 %	0.0 %	<0.001
Specificity	100.0 %	100.0 %	1.00
Subgroup 3			
Sensitivity	25.0 %	28.6 %	0.67
Specificity	100.0 %	100.0 %	1.00

**Supplemental Table 2: Diagnostic Accuracy 9 – 30mm**		
	Model 1	Model 2
Subgroup 4 (n = 123)		
Youden-Index	0.16	0.68
Cutoff value	74.09	0.81
Sensitivity	56.3 %	71.8 %
Specificity	60.0 %	35.0 %
FNR	8.8 %	9.3 %
FPR	83.3 %	71.4 %

FNR = False-positive rate; FPR = False-negative rate

**Supplemental Table 3: Regression analyses, Classification vs. no classification**				
Variable	Model 1		Model 2	
	Univariate Hazard Ratio	p – value	Univariable Hazard Ratio	p – value
Sex	1.02	0.96	0.62	0.21
Age	0.62	0.24	0.53	0.11
Slice thickness	0.67	0.37	0.53	0.11
Type of sample	2.15	1.5	1.27	0.68
CT location	1.25	0.62	0.57	0.39
Use of contrast media	1.44	0.35	0.8	0.54
CT type (breath-hold vs breathing)	0.8	0.67	1.68	0.32

Categorial variables were encoded as dummy variables.
